# THE RELATIONSHIP BETWEEN PHYSICIAN VISITS AND ADVANCE CARE PLANNING

**DOI:** 10.1093/geroni/igac059.2516

**Published:** 2022-12-20

**Authors:** YuHsuan (Olivia) Wang, Susan Enguidanos

**Affiliations:** USC, Los Angeles, California, United States; University of Southern California, Los Angeles, California, United States

## Abstract

Some studies have shown that advance care planning (ACP), discussions about future care given a serious illness, are associated with improved quality of death and better end-of-life care. Studies also have found that physicians play an important role in ACP. In fact, in 2016 CMS began reimbursing physicians for ACP discussions. However, little is known about the relationship between the number of physician visits and engaging in ACP . This study investigated the association between outpatient physician visits and ACP engagement. Only respondents over 65 were included in our study. We conducted logistic regressions using the 2016 Health and Retirement Study. We used ACP engagement (n=9838), advance directive (AD; eg, living will) completion (n=9746), and healthcare power of attorney (e.g., proxy) assignment (n=9724) as outcome variables. In addition to the frequency of physician visits, we controlled for basic demographics (age, gender, marital status, race, and socioeconomic status), number of chronic conditions, and self-rated health in our models. For each additional physician visit, the probability of having an ACP conversation, (p<.001), AD completion (p<.001), and healthcare proxy assignment (p<.001) increased by 1.6%,1.4%, and 1.9% respectively after controlling for covariates. Number of chronic conditions also was independently and positively associated with ACP-related activities. Additionally, greater perceived health was associated with higher odds of AD completion (OR= 1.08, p<.05) and healthcare proxy assignment (OR= 1.07, p<.05). This study found that frequent encounters with physicians are associated with higher rates of ACP engagement even after controlling for health conditions.

